# Apoptosis‐Inducing and Cytotoxic Effects of the *Daphne mucronata* Extract and Its Bioactive Compound Gnidilatimonoein in Liver Cancer Cells

**DOI:** 10.1155/bmri/3638482

**Published:** 2026-11-14

**Authors:** Sevda Zarei, Manijeh Mianabadi, Mahnaz Khalafi

**Affiliations:** ^1^ Department of Biology Faculty of Sciences Golestan University Gorgan Iran gu.ac.ir; ^2^ Department of Biology Faculty of Sciences University of Guilan Rasht Iran guilan.ac.ir; ^3^ Department of Statistics Faculty of Sciences Golestan University Gorgan Iran gu.ac.ir

**Keywords:** apoptosis, Bax/Bcl-2 ratio, caspase-3, *Daphne mucronata*, gnidilatimonoein, natural products

## Abstract

Hepatocellular carcinoma (HCC) continues to rank among the leading causes of cancer‐related death worldwide, and the modest efficacy together with the systemic toxicity of conventional chemotherapeutic regimens continues to constrain treatment outcomes. Identifying safer, more selective anticancer agents therefore remains a priority in toxicological and pharmacological research. *Daphne mucronata*, a medicinal plant native to Iran, has previously demonstrated anti‐inflammatory and cytotoxic properties, yet the anticancer potential of the plant and its bioactive constituents in liver cancer has not been thoroughly characterized. In the present study, we examined the anticancer activity of *D*. *mucronata* extract and its purified diterpenoid constituent, gnidilatimonoein (GND), using HCC models. GND was isolated through column chromatography and further purified by high‐performance liquid chromatography. Cytotoxicity and IC_50_ values were determined in human HepG_2_ and murine Hepa1‐6 liver cancer cell lines, as well as normal human hepatocytes (THLE‐2), using the MTT assay. Apoptotic activity was evaluated through annexin V/PI flow cytometry, caspase‐3 activity assays, nitric oxide (NO) measurement, liver enzyme profiling, and Western blot analysis of Bax, Bcl‐2, P53, and caspase‐3 expression. Both the crude extract and purified GND significantly suppressed cancer cell proliferation in a dose‐dependent manner (*p* < 0.0001), with IC_50_ values of 1.36 mg/mL (HepG_2_) and 1.23 mg/mL (Hepa1‐6) for the extract, and 3.439 *μ*g/mL (HepG_2_) and 34.23 ng/mL (Hepa1‐6) for GND. Notably, GND exhibited pronounced selectivity for malignant cells over normal hepatocytes and showed changes in apoptosis‐related protein expression (elevated Bax, P53, and caspase‐3, and decreased Bcl‐2) consistent with engagement of the intrinsic mitochondrial apoptotic pathway. Together, these findings position GND as a promising plant‐derived candidate for HCC therapy, warranting further investigation into its in vivo efficacy and translational potential.

## 1. Introduction

Liver cancer, particularly HCC, is a leading cause of cancer‐related mortality worldwide and the fifth most prevalent cancer in the United States, with a disproportionately high burden in developing countries [1]. Its pathogenesis is multifactorial, involving chronic hepatitis B/C infection, nonalcoholic fatty liver disease (NAFLD), alcohol‐related cirrhosis, obesity, diabetes, and other risk factors [2]. Prognosis remains generally poor, as most patients present at an advanced stage; only 5%–15% of noncirrhotic patients are eligible for curative resection or transplantation [3]. Transarterial chemoembolization (TACE) improves 2‐year survival in advanced disease, and sorafenib remains the mainstay systemic therapy for late‐stage HCC, but its clinical benefit is limited by acquired resistance in roughly one‐third of patients within 6 months and by considerable systemic toxicity [4, 5]. These limitations underscore the urgent need for safer, more selective therapeutic strategies.

In this context, natural compounds derived from medicinal plants have gained increasing attention as potential adjuncts or alternatives to conventional therapies. Plants of the *Daphne* genus, belonging to the Thymelaeaceae family, are known for their rich composition of bioactive diterpenoids characterized by orthoester groups and diverse tricyclic ring structures. To date, approximately 135 distinct diterpenoid compounds have been characterized from various species within this genus, which can be categorized into three principal structural classes [6]. Emerging research highlights the diverse pharmacological activities of these compounds, including anticancer, anti‐inflammatory, anti‐HIV, antifungal, neurotrophic, and cholesterol‐lowering effects [7]. Among the *Daphne* species, *Daphne mucronata* has been traditionally used in Chinese and Iranian medicine for its therapeutic properties [8]. GND, a diterpene ester with a daphnane‐type orthoester skeleton isolated from *D*. *mucronata*, was originally characterized for its ability to inhibit substrate–tumor cell adhesion in human platelets and WEHI‐164 cells [9, 10]. Subsequent reports demonstrated that GND induces monocytic differentiation and apoptosis in acute leukemia cell lines (KG1, NB4, U937, and HL‐60), with IC_50_ values in the low‐micromolar range [11, 12], acts as an IMPDH inhibitor that depletes intracellular GTP pools in K562 cells [13], and suppresses NO production in LPS‐stimulated glial cells [14]. Related *Daphne*‐derived diterpenoids and polyphenols have also shown antiangiogenic activity in other tumor systems [15]. However, these findings derive almost exclusively from hematologic malignancies or nonhepatic models; to date, no study has examined GND′s cytotoxic and apoptotic mechanism in a solid epithelial tumor such as hepatocellular carcinoma, nor compared its selectivity margin against normal hepatocytes or benchmarked its efficacy against a clinically used HCC agent such as sorafenib. Whether GND′s proapoptotic activity extends to solid hepatic tumors, and whether it retains selectivity toward malignant over normal human hepatocytes while matching or exceeding the efficacy of standard chemotherapeutics such as sorafenib, has not previously been addressed.

Apoptosis, or programmed cell death, represents a pivotal mechanism for maintaining tissue homeostasis and is a key target in cancer therapy. Dysregulation of apoptotic pathways—particularly the balance between proapoptotic proteins (e.g., Bax) and antiapoptotic proteins (e.g., Bcl‐2)—contributes significantly to tumor initiation and progression [16–18]. The ability of natural compounds to restore this balance and selectively induce apoptosis in cancer cells has made them promising candidates for anticancer drug development.

Cell lines have become indispensable tools in cancer research, offering a cost‐effective and ethical means to study tumor biology and therapeutic responses. Among the most commonly used liver cancer cell lines are human HepG_2_ and murine Hepa1‐6 cells [19, 20]. HepG_2_ cells are derived from a well‐differentiated human liver carcinoma and are widely utilized due to their ability to retain many liver‐specific functions, making them a suitable model for studying human liver cancer. In contrast, Hepa1‐6 cells, derived from a mouse hepatic carcinoma, are frequently used to evaluate the effects of novel therapies in a murine model, providing insights complementary to human studies.

Building on this rationale, the present study seeks to address this gap by evaluating the therapeutic potential of *D*. *mucronata* and GND as safe, plant‐derived alternatives capable of overcoming the limitations of current chemotherapeutics such as sorafenib. By elucidating their mechanisms of apoptosis induction and regulation of apoptotic signaling, this work provides a scientific foundation for the development of novel, natural treatments for hepatocellular carcinoma.

This study is aimed at investigating the cytotoxic and proapoptotic effects of *D*. *mucronata* extract and its isolated compound GND on human (HepG_2_) and murine (Hepa 1‐6) liver cancer cell lines. The study specifically examines their ability to reduce NO production, alter key apoptotic protein expressions (Bax, Bcl‐2, P53, and caspase‐3), and induce apoptosis through intrinsic signaling pathways.

It is hypothesized that *D*. *mucronata* extract and its bioactive compound GND exert anticancer effects on liver cancer cells by suppressing cell proliferation and activating intrinsic apoptotic pathways—characterized by increased caspase‐3 activity, upregulated Bax and P53 expression, and downregulated Bcl‐2 expression—without inducing cytotoxicity in normal liver cells.

## 2. Materials and Methods

### 2.1. Extraction and Purification of GND From *D*. *mucronata*

The plant material of *D*. *mucronata* was collected from Yasuj Forest Park (51°41 ^′^–51°48 ^′^ E, 30°23 ^′^–30°30 ^′^ N), Iran (Supplementary Figure 1). The identity was formally authenticated by Dr. Jafari, Department of Botany, Yasuj University, and a voucher specimen (No. 6035) was deposited in the publicly available Herbarium of Yasuj University (YU‐Herb), Yasuj, Iran.

Collection was performed with permission from Yasuj Forest Park authorities, under the Iranian Natural Resources and Watershed Management Organization (NRWMO), in compliance with national regulations on public land sampling (Article 12, Forest and Range Law of Iran, 1964, amended 2018). Appropriate licenses were obtained from the NRWMO Kohgiluyeh and Boyer‐Ahmad Provincial Office. No private land was involved, and the study adheres to ethical guidelines of the Convention on Biological Diversity, ratified by Iran.

For extract preparation, 50 g of powdered *D*. *mucronata* leaf material was mixed with 100 mL of 70% ethanol in a flask and agitated on a shaker for 48 h at room temperature. The mixture was then filtered twice, and the filtrate was stored at 4°C. The residue remaining on the filter was returned to the flask, mixed with an additional 100 mL of 70% ethanol, and agitated on a shaker for 24 h under the same conditions. The resulting solution was filtered, and the filtrate was combined with the previously stored filtrate at 4°C. The combined extracts were concentrated to dryness using a rotary evaporator at 40°C, yielding 1 g of dry crude extract (2% *w*/*w* relative to the starting leaf material). The resulting residue was dissolved in water, and following centrifugation, the supernatant was sterile‐filtered through a 0.22‐*μ*m syringe filter under a laminar flow hood, yielding a final extract concentration of 1 mg/mL in water. This solution was filtered once more and loaded onto the chromatography column to isolate GND.

GND was purified following Mianabadi et al. [9, 10] using column chromatography with diethyl ether (Sigma‐Aldrich, United Kingdom), followed by high‐performance liquid chromatography (HPLC). The yield of purified GND was 0.0171 g per gram of dry crude extract (1.71% *w*/*w*), corresponding to 0.0342% (*w*/*w*) relative to the original dry leaf material. HPLC analysis of the purified compound (Agilent 1260 system; Zorbax C18 column; mobile phase of deionized water and acetonitrile under the gradient program detailed in Supplementary Table 2; flow rate of 1 mL/min; detection wavelength of 326 nm) showed a single, well‐resolved peak at a retention time of 16.4 min with no detectable coeluting impurity peaks, indicating a purity of > 95% (Supplementary Figure 1c). Detailed purification procedures, including chromatographic column specifications and HPLC gradient conditions, are provided in supplementary data (Section 1.1). The chemical structure of GND, a daphnane‐type diterpene orthoester, is presented in Supplementary Figure 1e.

### 2.2. Cell Culture

Human hepatocellular carcinoma (HepG_2_), murine hepatocarcinoma (Hepa1‐6), and normal human liver (THLE‐2) cell lines were obtained from the National Cell Bank of Iran (NCBI), Pasteur Institute of Iran. All cell culture reagents were supplied by Sigma‐Aldrich (United Kingdom). Cells were cultured in Dulbecco′s Modified Eagle Medium (DMEM) supplemented with 10% fetal bovine serum (FBS) (20% for THLE‐2 cells), 100‐U/mL penicillin, 100‐*μ*g/mL streptomycin, and 2‐mM L‐glutamine. Cultures were maintained at 37°C in a humidified atmosphere containing 5% CO_2_.

The culture medium was refreshed every 24 h to ensure optimal cell growth. When cells reached approximately 80%–90% confluence (after 3–5 days), they were subcultured using 0.25% trypsin‐EDTA. Detached cells were centrifuged at 3000 rpm for 5 min, washed twice with phosphate‐buffered saline (PBS), and resuspended in fresh medium for subsequent experiments [21].

### 2.3. Calculation of the IC_50_ and Selectivity Index (SI)

HepG_2_, Hepa1‐6, and THLE‐2 cells were seeded at a density of 5 × 10^5^ cells/mL into 96‐well plates. The cells were incubated at 37°C in a humidified atmosphere containing 5% CO_2_ and 95% relative humidity, using culture medium composed of 90% DMEM, 10% FBS, and 1% penicillin–streptomycin (100 U/mL). After 24 h of incubation, the medium was replaced with DMEM containing 1% FBS.

HepG_2_ and THLE‐2 cells were treated with various concentrations of *D*. *mucronata* extract (0–2.5 mg/mL) and GND (0–4.275 *μ*g/mL) for 24 h. Sorafenib (Sigma‐Aldrich, United States) was prepared by dissolving the powder in 0.1% dimethyl sulfoxide (DMSO), a concentration verified to be nontoxic to cells [22, 23]. HepG_2_ cells were then treated with sorafenib at concentrations ranging from 0 to 64 *μ*g/mL. Similarly, Hepa1‐6 cells were exposed to *D*. *mucronata* extract (0–1.625 mg/mL) and GND (0–38.475 ng/mL) under identical conditions. Untreated cells served as the control group.

Cell viability, proliferation, and cytotoxicity were assessed using the MTT assay [23, 24]. All experiments were performed under aseptic conditions, with each concentration tested in triplicate at a final volume of 200 *μ*L per well. An initial broad concentration range was screened, after which the optimal, minimally toxic range was selected for further analysis. To determine the IC_50_ values for *D*. *mucronata* and GND, the MTT assay was performed based on the enzymatic reduction of the tetrazolium salt MTT to an insoluble purple formazan product, which was quantified spectrophotometrically [22, 25, 26]. After treatment, the supernatant in each well was removed—retained for NO analysis—and replaced with 90 *μ*L of serum‐free medium and 10 *μ*L of MTT solution (5 mg/mL; Sigma‐Aldrich). The plates were incubated at 37°C for approximately 2.5–4 h. Formazan crystals were then solubilized by adding 100 *μ*L of DMSO and incubating for 30 min. Absorbance was measured at 570 nm using a microplate reader (Bio‐Rad Laboratories, United States).

The percentage of growth inhibition and cell viability was calculated using the following equations:

Percentage of inhibitory= 1−OD treatmentOD control×100%,

Cell viability= OD treatmentOD control×100%.

Dose–response data were plotted, and IC_50_ values were determined from the regression line described by the equation *y* = ax + *b* [24, 27].

To assess the preferential cytotoxicity of *D*. *mucronata* extract and GND toward malignant over normal hepatocytes, the SI was calculated as follows:

SI=IC50 normal cell line;THLE−2IC50 cancer cell line,

where higher SI values indicate greater selectivity for cancer cells [15]. Because neither compound reduced THLE‐2 cell viability by 50% within the tested concentration range, IC_50_ (THLE‐2) could not be directly determined; in such cases, the highest tested concentration was used as a conservative lower bound estimate of IC_50_ (THLE‐2), yielding minimum SI values. Where the dose–response relationship in THLE‐2 cells remained statistically significant and well fit by linear regression across the tested range, IC_50_ (THLE‐2) was additionally estimated by extrapolation from the regression equation, providing a complementary exploratory SI estimate.

### 2.4. Measuring NO

NO levels were quantified using the Griess reagent assay (Sigma‐Aldrich, United States). The reagent was prepared by dissolving 0.25 g of Griess powder in 25 mL of double‐distilled water. After treatment, the culture supernatants collected from the experimental wells (as described in Section 2.3) were used for analysis. For each sample, 280 *μ*L of supernatant was transferred into a 96‐well microplate, followed by the addition of 20 *μ*L of Griess reagent. A blank control containing 280 *μ*L of DMEM and 20 *μ*L of reagent was also included. The mixtures were incubated at room temperature for 30 min in the dark to allow color development.

The absorbance of each well was then measured at 570 nm using a microplate reader (Bio‐Rad Laboratories, United States). Nitrite concentrations, representing NO production, were determined from a standard calibration curve generated using sodium nitrite (NaNO_2_) (Merck, Germany) at concentrations ranging from 0 to 60 *μ*M [28].

### 2.5. Treatment Groups

HepG_2_ and Hepa1‐6 cells were seeded separately at a density of 6 × 10^5^ cells/mL in six‐well plates and incubated at 37°C with 5% CO_2_ and 95% relative humidity. After 24 h, the medium was replaced with DMEM containing 1% FBS to minimize cell proliferation and synchronize the cells prior to treatment. The cells were divided into five experimental groups as follows:

1. Control group: untreated cells,
2. *D*. *mucronata* (2/3 IC_50_): cells treated with two‐thirds of the IC_50_ concentration of *D*. *mucronata* extract,
3. *D*. *mucronata* (IC_50_): cells treated with the IC_50_ concentration of *D*. *mucronata* extract,
4. GND (2/3 IC_50_): cells treated with two‐thirds of the IC_50_ concentration of GND, and
5. GND (IC_50_): cells treated with the IC_50_ concentration of GND.

Each treatment was performed in triplicate for 24 h, with a final volume of 2 mL per well. Each of the three replicate wells per treatment group was processed and lysed independently, yielding three independent samples per group that were each analyzed separately in the downstream assays described below. All procedures were conducted under aseptic conditions to prevent contamination. After treatment, the cells were collected for subsequent biochemical and molecular analyses. Total protein concentrations in each group were determined using the Bradford assay [29, 30], with bovine serum albumin (BSA) (Sigma‐Aldrich, United States) used as the standard reference (Supplementary Figure 2).

### 2.6. Estimation of Caspase‐3 Activity

Caspase‐3 activity was evaluated in HepG_2_ and Hepa1‐6 cells following treatment with *D*. *mucronata* extract GND. The analysis was performed using a commercial enzyme‐linked immunosorbent assay (ELISA) kit (Thermo Fisher Scientific, United States) according to the manufacturer′s protocol. Briefly, after 24 h of treatment, cells were harvested and lysed using the lysis buffer provided in the kit. The lysates were centrifuged at 10,000 rpm for 10 min at 4°C to remove cellular debris, and the resulting supernatants were collected for analysis. Protein concentrations were determined using the Bradford assay to ensure equal protein loading across all samples. Caspase‐3 activity was quantified colorimetrically by measuring the absorbance of the reaction product at 405 nm using a microplate reader (Bio‐Rad Laboratories, United States). Enzyme activity was expressed as fold change relative to the control group [30, 31]. The ELISA‐based caspase‐3 activity kit used in this study (Thermo Fisher Scientific) is validated by the manufacturer against a reference recombinant caspase‐3 standard curve, ensuring assay sensitivity and specificity; a chemically induced positive apoptotic control (e.g., staurosporine‐ or doxorubicin‐treated cells) was not included as a parallel benchmark in the present experiments.

### 2.7. Biochemical Assays

Following treatment, HepG_2_ and Hepa1‐6 cells were harvested, washed twice with PBS, and lysed to assess intracellular enzyme activities. The activities of AST, ALT, ALP, LDH, and GGT were measured to evaluate the biochemical effects of *D*. *mucronata* extract and GND on cellular integrity and metabolic function. Cell lysis was performed using a suitable lysis buffer according to the manufacturer′s instructions, followed by centrifugation at 10,000 rpm for 10 min at 4°C to remove cellular debris. The resulting supernatant (cell lysate) was then used for enzyme assays.

Enzyme activities were determined using commercial diagnostic kits (Pars Azmoon Co., Tehran, Iran) following the provided protocols. Each test was performed in triplicate to ensure data reliability. Enzyme activities were expressed as units per liter (U/L) and normalized to the total protein concentration in each sample, quantified using the Bradford assay. These biochemical markers served as indicators of hepatocellular viability, membrane integrity, and metabolic activity [32, 33].

Unlike their conventional clinical use as serum biomarkers—where enzyme leakage from damaged hepatocytes into the circulation reflects tissue injury—these enzymes were measured here in intracellular lysates to assess retained cytosolic enzymatic content following treatment. A treatment‐associated decline in intracellular enzyme activity is consistent with two nonmutually exclusive mechanisms: (i) loss of plasma membrane integrity permitting enzyme leakage into the extracellular medium, the same underlying principle exploited by enzyme‐release cytotoxicity assays such as the LDH‐release assay [34, 35], and (ii) reduced overall cellular metabolic and biosynthetic activity in cells undergoing cytotoxic stress and apoptosis. This intracellular enzyme‐activity approach has been previously applied by our group as an in vitro correlate of hepatocellular injury and metabolic dysfunction in other cell‐based toxicological models [21, 25, 32, 36].

### 2.8. Western Blot Analysis

Western blot analysis was performed to evaluate the expression levels of apoptosis‐related proteins in HepG_2_ cells following treatment with GND. The HepG_2_ cells were categorized into two experimental groups: (1) control group: untreated cells and (2) GND‐treated group: cells exposed to GND at a concentration of 3.439 *μ*g/mL. After 24 h of treatment, cells were harvested and washed twice with ice‐cold PBS. Cell lysis was carried out using radioimmunoprecipitation assay (RIPA) buffer containing 1% phenylmethylsulfonyl fluoride (PMSF) to inhibit protease activity. The lysates were incubated on ice for 30 min and then centrifuged at 12,000 rpm for 10 min at 4°C to remove cellular debris. The resulting supernatants, containing soluble proteins, were collected for further analysis. Total protein concentrations were quantified using the bicinchoninic acid (BCA) assay kit (Thermo Fisher Scientific, United States). Equal amounts of protein (25 *μ*g per sample) were mixed with loading buffer, denatured by heating at 95°C for 5 min, and separated by sodium dodecyl sulfate‐polyacrylamide gel electrophoresis (SDS‐PAGE) using 12% resolving and 5% stacking gels. Proteins were then transferred onto polyvinylidene fluoride (PVDF) membranes (Millipore, United States) using a semidry transfer system.

The membranes were blocked with 5% nonfat dry milk in Tris‐buffered saline with 0.1% Tween‐20 (TBST) for 1 h at room temperature, followed by overnight incubation at 4°C with primary antibodies against Bax, Bcl‐2, P53, and caspase‐3 (Santa Cruz Biotechnology, United States). Glyceraldehyde‐3‐phosphate dehydrogenase (GAPDH) was used as the internal loading control.

After washing, membranes were incubated with horseradish peroxidase (HRP)–conjugated secondary anti‐mouse IgG antibodies (Sigma‐Aldrich, United States) for 1 h at room temperature. Protein bands were visualized using an enhanced chemiluminescence (ECL) detection kit (Bio‐Rad Laboratories, United States) and imaged using the ChemiDoc XRS+ system (Bio‐Rad, United States). The relative intensities of the target proteins were quantified by densitometric analysis and normalized to GAPDH expression [36, 37].

### 2.9. Apoptosis Assay via Flow Cytometry

Apoptotic cell death in HepG_2_ cells was evaluated using the annexin V–fluorescein isothiocyanate (FITC)/propidium iodide (PI) double‐staining method with a commercial apoptosis detection kit (Elabscience, China). For the HepG_2_ cell line, three experimental groups were analyzed: (1) control group: untreated cells, (2) *D*. *mucronata* group: cells treated with *D*. *mucronata* extract at 1.36 mg/mL, and (3) GND group: cells treated with GND at 3.439 *μ*g/mL.

Following treatment for 24 h, both adherent and floating cells were collected to ensure comprehensive recovery. The cells were washed twice with ice‐cold PBS and resuspended in 500 *μ*L of binding buffer at a final concentration of approximately 1 × 10^6^ cells/mL.

Subsequently, 3 *μ*L of annexin V‐FITC and 3 *μ*L of PI (1 mg/mL) were added to each sample. The mixtures were gently vortexed and incubated for 20 min at room temperature in the dark to prevent fluorochrome photobleaching. After incubation, samples were immediately analyzed using a FACSCalibur flow cytometer (Becton Dickinson, United States). Data were processed using FlowJo software (Version 7.6.1; FlowJo LLC, Ashland, Oregon). Based on the fluorescence profiles, cells were classified as follows: viable cells: annexin V^−^/PI^−^, early apoptotic cells: annexin V^+^/PI^−^, late apoptotic cells: annexin V^+^/PI^+^, and necrotic cells: annexin V^−^/PI^+^. The percentage of apoptotic and necrotic cells was determined using quadrant analysis to compare the effects of treatments among groups [38]. The annexin V‐FITC/PI kit (Elabscience) includes internal manufacturer‐provided positive and negative staining controls used to calibrate compensation and gating; however, a cell‐based chemotherapeutic positive apoptotic control was not run in parallel with the experimental groups.

### 2.10. Statistical Analysis

For the MTT‐based IC_50_ determination assays, each concentration was tested in three replicate wells from the same cell culture on the same day (technical replicates). For the treatment‐group experiments (Section 2.5), each treatment group (control, 2/3 IC_50_, and IC_50_) was established in three independently treated and separately processed wells; the resulting samples were each analyzed individually in the downstream caspase‐3 activity, biochemical enzyme, Western blot, and flow cytometry assays, and results are expressed as the mean ± SEM of these three independently processed replicates. Statistical analyses were performed using GraphPad Prism software (Version 9.1) and SPSS (Version 23). Data normality and homogeneity of variance were verified using the Kolmogorov–Smirnov test. Differences among treatment groups were evaluated using one‐way analysis of variance (ANOVA) followed by Tukey′s honestly significant difference (HSD) post hoc test for pairwise comparisons. In cases where only two groups were compared, a Student′s *t*‐test was applied. The level of statistical significance was set at  ^∗∗^ *p* < 0.01 and  ^∗∗∗∗^ *p* < 0.0001. All results were validated by repeating experiments independently to ensure reliability and reproducibility.

## 3. Results

### 3.1. Cytotoxic Effects of *D*. *mucronata* and GND

The cytotoxic and antiproliferative effects of *D*. *mucronata* extract and its purified compound, GND, were evaluated in HepG_2_, Hepa1‐6, and THLE‐2 cells using the MTT assay (Figure 1; *p* < 0.0001). As shown in Figure 1a,b, the *D*. *mucronata* extract significantly inhibited cell proliferation in both HepG_2_ and Hepa1‐6 cell lines in a dose‐dependent manner, which was accompanied by a corresponding increase in cell death. The IC_50_ values for the extract were 1.36 and 1.23 mg/mL for HepG_2_ and Hepa1‐6 cells, respectively.

**Figure 1:**
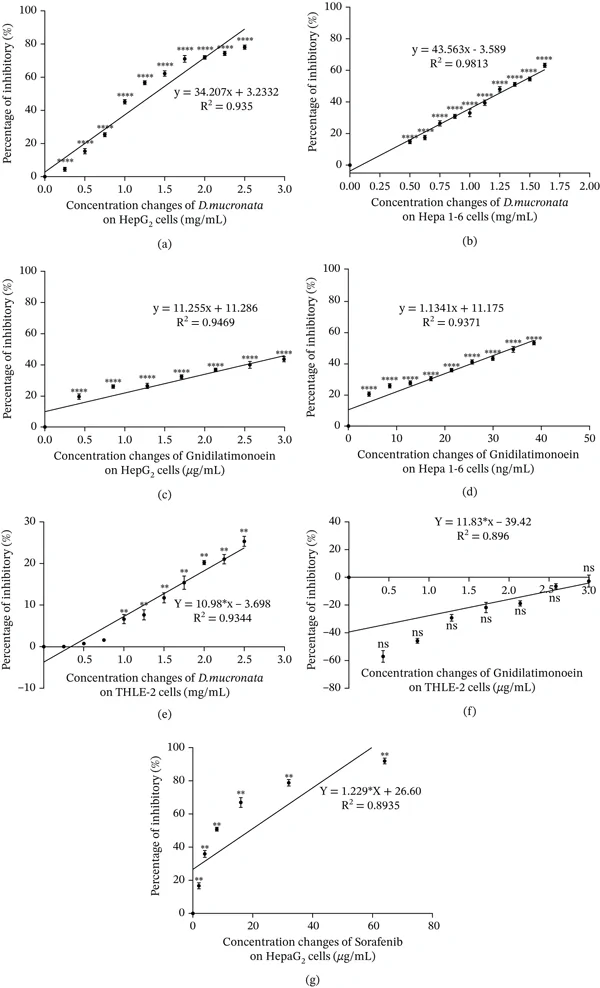
Cell viability and determination of IC_50_ values. IC_50_ values for *D*. *mucronata* and GND in HepG_2_, Hepa1‐6, and THLE‐2 cells were determined (*n* = 3; mean ± SEM). (a) The IC_50_ of *D*. *mucronata* in HepG_2_ cells was 1.36 mg/mL. (b) In Hepa1‐6 cells, the IC_50_ for *D*. *mucronata* was 1.23 mg/mL, with a clear dose‐dependent increase in growth inhibition. (c) The IC_50_ for GND in HepG_2_ cells was 3.439 *μ*g/mL. (d) In Hepa1‐6 cells, the IC_50_ of GND was 34.234 ng/mL, also showing dose‐dependent growth inhibition. (e) *D*. *mucronata* exhibited cytotoxicity toward THLE‐2 cells only at higher concentrations, with 2.5 mg/mL causing approximately a 24% reduction in viability. (f) GND did not induce significant cytotoxicity in THLE‐2 cells at any tested concentration. (g) Sorafenib treatment in HepG_2_ cells resulted in a dose‐dependent reduction in cell proliferation, with an IC_50_ value of 19.03 *μ*g/mL. Linear regression analysis was used to examine the relationship between concentration (*x*) and cell viability (*y*), described by the equation *y* = *a* *x* + *b*. The slope (*a*) and intercept (*b*) represent the regression line parameters. The coefficient of determination (*R* ^2^) was calculated to assess the model′s goodness of fit; higher *R* ^2^ values indicate that the model effectively explains the observed variance and accurately represents the data. Each treatment was tested in triplicate to ensure reliability. Statistical significance among groups was determined by a one‐sample *t*‐test (*p* < 0.0001, *p* < 0.01; ns: not significant). Abbreviation: GND, gnidilatimonoein.

Similarly, GND treatment induced a marked, dose‐dependent reduction in cell viability in both cancer cell lines (*p* < 0.0001). The IC_50_ values for GND were 3.439 *μ*g/mL (5.1 *μ*M) for HepG_2_ cells (Figure 1c) and 34.23 ng/mL (51.71 nM) for Hepa1‐6 cells (Figure 1d). In contrast, although the *D*. *mucronata* extract showed only mild cytotoxicity toward THLE‐2 cells at higher concentrations—reducing cell viability by approximately 24% at 2.5 mg/mL (Figure 1e)—GND did not exhibit significant cytotoxic effects in THLE‐2 cells at any of the tested concentrations (Figure 1f). For comparison, sorafenib, a standard chemotherapeutic agent, also produced a dose‐dependent inhibition of HepG_2_ cell proliferation, with an IC_50_ value of 19.03 *μ*g/mL (40 *μ*M) (Figure 1g; *p* < 0.0001).

To further characterize this differential activity, the SI (SI = IC_50_ [THLE − 2]/IC_50_ [cancer cell line]) was calculated (Table 1). Because neither compound reduced THLE‐2 viability by 50% within the tested range, conservative lower bound SI values were > 1.84 (HepG_2_) and > 2.03 (Hepa1‐6) for the extract, and > 1.24 (HepG_2_) and > 124.9 (Hepa1‐6) for GND. As the dose–response relationship of the extract in THLE‐2 cells remained statistically significant across the tested range (Figure 1e; *R* ^2^ = 0.934), IC_50_ (THLE‐2) was additionally estimated by extrapolation, yielding exploratory SI values of ~3.60 (HepG_2_) and ~3.98 (Hepa1‐6). This extrapolation was not applied to GND, as no significant cytotoxicity trend was observed in THLE‐2 cells at any tested concentration (Figure 1f).

**Table 1:** SI calculation.

Treatment	Cancer cells	IC_50_ (cancer)	IC_50_ (THLE‐2)	SI	Basis
*D*. *mucronata*	HepG_2_	1.36 mg/mL	> 2.5 mg/mL	> 1.84	Conservative (measured)^a^
*D*. *mucronata*	HepG_2_	1.36 mg/mL	~4.89 mg/mL	~3.60	Exploratory (extrapolated)^b^
*D*. *mucronata*	Hepa1‐6	1.23 mg/mL	> 2.5 mg/mL	> 2.03	Conservative (measured)^a^
*D*. *mucronata*	Hepa1‐6	1.23 mg/mL	~4.89 mg/mL	~3.98	Exploratory (extrapolated)^b^
GND	HepG_2_	3.439 *μ*g/mL	> 4.275 *μ*g/mL	> 1.24	Conservative (measured)^c^
GND	Hepa1‐6	0.03423 *μ*g/mL	> 4.275 *μ*g/mL	> 124.9	Conservative; cross‐species

^a^Highest tested concentration in THLE‐2 cells used as a conservative lower bound, since IC_50_ was not reached (2.5 mg/mL for the extract; ~24% inhibition observed).

^b^Extrapolated from the significant linear regression for *D*. *mucronata* extract in THLE‐2 cells (Figure 1e); reported as an exploratory estimate beyond the tested range, not a directly measured value.

^c^GND produced no statistically significant cytotoxicity in THLE‐2 cells at any tested concentration (all points ns; Figure 1f); extrapolation was therefore not performed, and only the conservative lower bound SI is reported.

Collectively, these results indicate that both the *D*. *mucronata* extract and GND effectively suppress liver cancer cell proliferation while exerting minimal cytotoxicity toward normal liver cells. Notably, GND displayed greater potency than the crude extract in both cancer cell lines, underscoring its potential as a promising natural anticancer candidate.

### 3.2. Effects of *D*. *mucronata* Extract and GND on NO Levels

To assess the impact of *D*. *mucronata* extract and GND on NO production, NO concentrations were measured in the culture medium of HepG_2_ and Hepa1‐6 cells after 24 h of treatment (Figure 2; *p* < 0.0001). The results demonstrated a significant reduction in NO levels in both HepG_2_ and Hepa1‐6 cells treated with *D*. *mucronata* extract and GND compared with the control group. In HepG_2_ cells, treatment with *D*. *mucronata* extract led to a marked decline in NO concentration at both IC_50_ and 2/3 IC_50_ doses (Figure 2a; *p* < 0.0001). Similarly, GND treatment produced a dose‐dependent decrease in NO levels (Figure 2b; *p* < 0.0001). A comparable pattern was observed in Hepa1‐6 cells (Figure 2c,d), where both *D*. *mucronata* extract and GND significantly suppressed NO production relative to untreated controls (*p* < 0.0001). Among the treatments, GND exhibited a stronger inhibitory effect on NO generation compared with the plant extract at equivalent concentrations.

**Figure 2:**
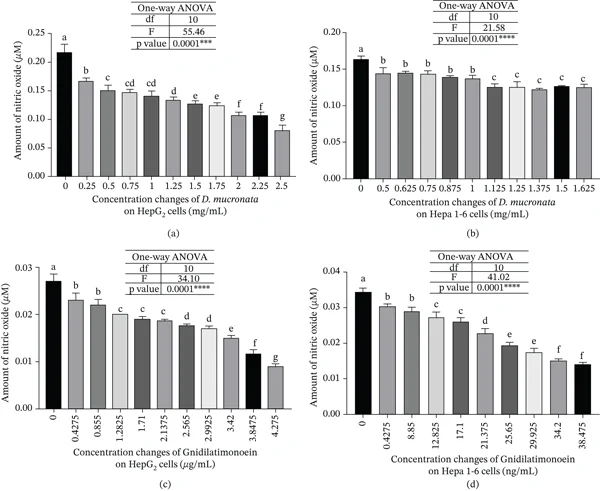
Analysis of NO concentrations in liver cancer cells treated with various concentrations of *D*. *mucronata* and GND. NO concentrations were quantified in HepG_2_ and Hepa1‐6 liver cancer cells treated with various concentrations of *D*. *mucronata* extract and its purified compound, GND (*n* = 3; mean ± SEM). (a) NO levels in HepG_2_ cells treated with *D*. *mucronata.* (b) NO levels in Hepa1‐6 cells treated with *D*. *mucronata.* (c) NO concentrations in HepG_2_ cells treated with GND. (d) NO concentrations in Hepa1‐6 cells treated with GND. Overall, NO production significantly decreased with increasing treatment concentrations across both cell lines. Each treatment was performed in triplicate to ensure reproducibility. Different letters within each column indicate statistically significant differences among treatment groups, as determined by Tukey′s HSD post hoc test (*p* < 0.0001). Abbreviations: NO, nitric oxide; GND, gnidilatimonoein.

These findings suggest that *D*. *mucronata* and its active component GND effectively reduce intracellular NO production in liver cancer cells, which may contribute to their antiproliferative and proapoptotic effects.

### 3.3. Caspase‐3 Activity

Our results demonstrated that treatment of HepG_2_ and Hepa1‐6 cells with *D*. *mucronata* extract and GND resulted in significant alterations in caspase‐3 activity (Figure 3). In HepG_2_ cells, the highest level of caspase‐3 activity was observed following treatment with *D*. *mucronata* at a concentration of 1.36 mg/mL, whereas the control group exhibited the lowest activity. Caspase‐3 activity was significantly elevated in all GND‐treated groups compared with the control group (Figure 3a; *p* < 0.0001). A similar trend was observed in Hepa1‐6 cells, where caspase‐3 activity peaked at a *D*. *mucronata* concentration of 1.23 mg/mL and was lowest in the control group. Notably, in Hepa1‐6 cells, caspase‐3 activity followed a consistent dose‐dependent pattern for both agents, with *D*. *mucronata* (0.82 and 1.23 mg/mL) and GND (22.82 and 34.23 ng/mL) each producing significant, concentration‐dependent increases relative to control (Figure 3b; *p* < 0.0001).

**Figure 3:**
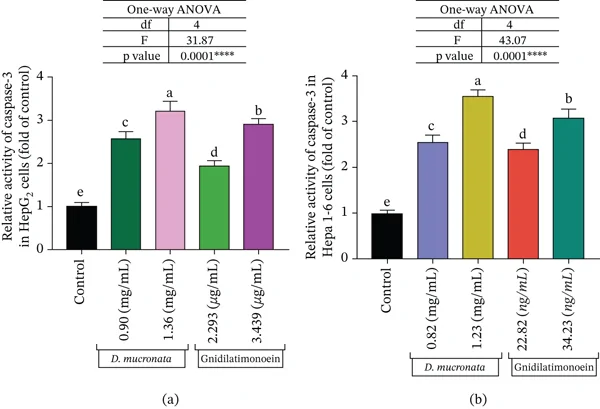
Effects of *D*. *mucronata* extract and GND on caspase‐3 activity in liver cancer cells. Caspase‐3 activity was measured in HepG_2_ and Hepa1‐6 liver cancer cells following treatment with varying concentrations of *D*. *mucronata* extract and GND (*n* = 3; mean ± SEM). (a) In HepG_2_ cells, five groups were analyzed: (1) control (untreated) cells, (2–3) *D*. *mucronata* at 0.90 and 1.36 mg/mL, and (4–5) GND at 2.293 and 3.439 *μ*g/mL. (b) In Hepa1‐6 cells, five groups were analyzed: (1) control (untreated) cells, (2–3) *D*. *mucronata* at 0.82 and 1.23 mg/mL, and (4–5) GND at 22.82 and 34.23 ng/mL. Caspase‐3 activity increased significantly in all treatment groups compared with the control, indicating activation of apoptotic pathways. Different letters within each column represent statistically significant differences among groups, as determined by Tukey′s HSD post hoc test (*p* < 0.0001). Abbreviation: GND, gnidilatimonoein.

These results suggest that both *D*. *mucronata* extract and GND can activate caspase‐3, a key executioner enzyme in the apoptotic pathway, thereby contributing to their proapoptotic effects in liver cancer cells.

### 3.4. Biochemical Parameters

Biochemical markers of liver function—including AST, ALT, ALP, LDH, and GGT—showed significant changes in HepG_2_ cells following treatment with *D*. *mucronata* extract and GND (Table 2; *p* < 0.01). Both treatment groups exhibited a consistent pattern of enzyme activity alterations. Notably, the control group demonstrated the highest levels of enzyme activity, while increasing concentrations of *D*. *mucronata* extract and GND led to a marked, dose‐dependent decrease in AST, ALT, ALP, LDH, and GGT activities (*p* < 0.01).

**Table 2:** Biochemical factors in HepG_2_ cells treated with *D. mucronata* and GND (*n* = 3; mean ± SEM; *p* < 0.01). Within each column, values not sharing a common superscript letter differ significantly (Tukey′s HSD post hoc test, *p* < 0.01).

Treatment groups		Analysis of Biochemical parameters (unit per g total protein)				
		AST (U/g)	ALT (U/g)	ALP (U/g)	LDH (U/g)	GGT (U/g)
*D*. *mucronata*	Control	3.22 ± 0.084^a^	2.58 ± 0.155^a^	2.02 ± 0.379^a^	6.90 ± 0.158^ab^	7.55 ± 0.620^a^
	0.90 (mg/mL)	2.92 ± 0.118^b^	2.34 ± 0.132^b^	1.53 ± 0.053^c^	6.87 ± 0.076^b^	5.04 ± 0.689^d^
	1.36 (mg/mL)	2.51 ± 0.173^c^	1.92 ± 0.239^c^	1.52 ± 0.188^c^	6.39 ± 0.221^c^	2.65 ± 0.328^f^

GND	Control	3.01 ± 0.084^ab^	2.63 ± 0.123^a^	2.08 ± 0.187^a^	6.99 ± 0.174^a^	7.42 ± 0.514^b^
	2.293 (*μ*g/mL)	2.31 ± 0.118^d^	1.95 ± 0.115^c^	1.62 ± 0.133^b^	5.93 ± 0.161^d^	6.09 ± 0.444^c^
	3.439 (*μ*g/mL)	2.29 ± 0.069^d^	1.75 ± 0.110^d^	1.59 ± 0.108^b^	5.83 ± 0.153^e^	4.63 ± 0.312^e^

Abbreviations: ALP, alkaline phosphatase; ALT, alanine aminotransferase; AST, aspartate aminotransferase; GGT, gamma glutamyl transferase; GND, gnidilatimonoein; LDH, lactate dehydrogenase.

A similar trend was observed in Hepa1‐6 cells (Table 3; *p* < 0.01). As in HepG_2_ cells, the control group generally exhibited the highest enzyme activities, and treatment with *D*. *mucronata* extract and GND significantly reduced most enzyme activities in a dose‐dependent manner. A modest exception was noted for ALP, which showed a slightly higher value at the higher *D*. *mucronata* concentration (1.23 mg/mL) than at the lower concentration (0.82 mg/mL), and for ALT, which showed a slightly higher value at the higher GND concentration (34.23 ng/mL) than at the lower concentration (22.82 ng/mL); both values nonetheless remained significantly lower than control.

**Table 3:** Biochemical factors in Hepa1‐6 cells treated with *D*. *mucronata* and GND (*n* = 3; mean ± SEM). Within each column, values not sharing a common superscript letter differ significantly (Tukey′s HSD post hoc test, *p* < 0.01).

Treatment groups		Analysis of Biochemical parameters (Unit per g total protein)				
		AST (U/g)	ALT (U/g)	ALP (U/g)	LDH (U/g)	GGT (U/g)
*D*. *mucronata*	Control	2.65 ± 0.036^ab^	2.36 ± 0.085^a^	2.31 ± 0.117^a^	5.09 ± 0.139^a^	6.81 ± 0.312^b^
	0.82 (mg/mL)	2.24 ± 0.177^b^	2.01 ± 0.172^c^	1.23 ± 0.076^e^	4.37 ± 0.187^b^	4.75 ± 0.564^c^
	1.23 (mg/mL)	1.86 ± 0.154^c^	1.81 ± 0.119^d^	1.32 ± 0.108^d^	3.49 ± 0.321^d^	2.39 ± 0.412^f^

GND	Control	2.88 ± 0.103^a^	2.13 ± 0.173^b^	2.19 ± 0.121^b^	4.99 ± 0.115^a^	7.03 ± 0.087^a^
	22.82 (ng/mL)	2.14 ± 0.121^b^	1.15 ± 0.145^f^	1.74 ± 0.146^c^	3.63 ± 0.165^c^	4.12 ± 0.124^d^
	34.23 (ng/mL)	1.78 ± 0.089^c^	1.25 ± 0.083^e^	1.37 ± 0.112^d^	2.06 ± 0.133^e^	3.68 ± 0.317^e^

Abbreviations: ALP, alkaline phosphatase; ALT, alanine aminotransferase; AST, aspartate aminotransferase; GGT, gamma glutamyl transferase; GND, gnidilatimonoein; LDH, lactate dehydrogenase.

Overall, these findings indicate that *D*. *mucronata* and GND can modulate liver enzyme activity in hepatocellular carcinoma cells, reflecting their potential to alter metabolic and membrane‐associated enzyme functions during treatment.

### 3.5. Expression of Apoptosis‐Related Proteins in GND‐Treated HepG_2_ Cells

GND was selected for Western blot analysis because earlier experimental results demonstrated that it exhibited greater potency and selectivity than the crude *D*. *mucronata* extract. Specifically, GND produced lower IC_50_ values in both HepG_2_ and Hepa1‐6 cells, induced stronger caspase‐3 activation, and showed negligible toxicity toward THLE‐2 cells. These properties made GND the most suitable candidate for detailed protein‐level analysis to elucidate the molecular mechanisms underlying its proapoptotic effects. Therefore, Western blot analysis was performed exclusively for the GND‐treated group to further elucidate its apoptotic mechanism in HepG_2_ cells.

The experiment included two groups: an untreated control group and a GND‐treated group (3.439 *μ*g/mL). The expression levels of proapoptotic proteins (Bax, P53, and caspase‐3) and the antiapoptotic protein (Bcl‐2) were evaluated using Western blotting (Figure 4). Original gel images are provided in the supplementary data (Supplementary Figures 3, 4, 5, 6, and 7). As shown in Figure 4a,b, treatment with GND led to a significant increase in Bax protein expression, which was approximately 3.12‐fold higher than in the control group (*p* < 0.0001). Similarly, P53 expression increased by 2.5‐fold following GND treatment (*p* < 0.0001). In contrast, the level of the antiapoptotic protein Bcl‐2 decreased markedly, showing a 0.346‐fold reduction compared with the control group (*p* < 0.0001). In addition, caspase‐3 was detected in both its inactive (procaspase‐3) and cleaved (active) forms. GND treatment reduced procaspase‐3 levels to approximately 0.5‐fold of control, while significantly increasing cleaved caspase‐3 expression by 4.08‐fold relative to the control group (*p* < 0.0001).

**Figure 4:**
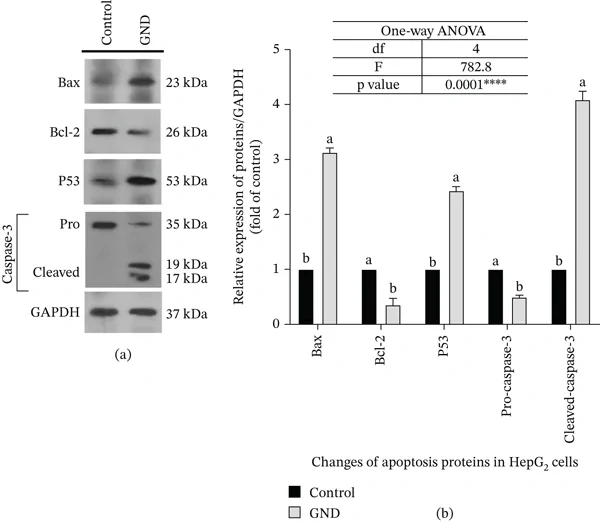
The relative expression levels of proapoptotic and antiapoptotic proteins assessed by Western blotting. The relative expression levels of proapoptotic and antiapoptotic proteins were evaluated in HepG_2_ cells following treatment with GND, 3.439 *μ*g/mL. Two groups were analyzed: (a) untreated control cells and (b) GND‐treated cells. The expression of Bax, P53, cleaved caspase‐3, and Bcl‐2 was assessed by Western blotting and normalized to GAPDH, which served as the internal loading control. Normalization was performed by adjusting each target protein′s band intensity relative to GAPDH to ensure accurate comparison among samples. Representative Western blot images are shown, with each panel depicting one of the analyzed proteins. Quantitative data are expressed as mean ± SEM. Statistical significance among the five relative‐expression fold‐changes was assessed by one‐way ANOVA; for each protein, the GND‐treated group was subsequently compared with its own untreated control, with different letters within each protein pair denoting a statistically significant difference (*p* < 0.0001). GND treatment markedly upregulated the proapoptotic proteins Bax, P53, and cleaved caspase‐3, while significantly downregulating the antiapoptotic protein Bcl‐2, resulting in a pronounced increase in the Bax/Bcl‐2 ratio, consistent with activation of the intrinsic apoptotic pathway. Abbreviations: GND, gnidilatimonoein; GAPDH, glyceraldehyde‐3‐phosphate dehydrogenase.

These findings indicate that GND modulates the expression of key apoptosis‐regulatory proteins in HepG_2_ cells, upregulating proapoptotic mediators (Bax, P53, and cleaved caspase‐3) and downregulating the antiapoptotic regulator Bcl‐2. This coordinated modulation of apoptotic signaling proteins is consistent with activation of the intrinsic (mitochondrial) apoptotic pathway, although direct evidence of mitochondrial outer membrane permeabilization—such as cytochrome c release or loss of mitochondrial membrane potential—was not assessed in this study.

### 3.6. Apoptotic/Necrotic Ratios in HepG_2_ Cells

Flow cytometry analysis was conducted exclusively on HepG_2_ cells because this human hepatocellular carcinoma line represents a clinically relevant model for studying the molecular mechanisms of liver cancer. HepG_2_ cells are well characterized and widely used in anticancer research due to their preserved metabolic and apoptotic signaling pathways, which closely reflect human liver tumor biology. In contrast, Hepa1‐6 cells, derived from murine liver carcinoma, were primarily used to confirm cytotoxic trends observed in human cells but are less suitable for detailed apoptotic profiling due to species‐specific differences in cell surface markers and intracellular signaling proteins. Thus, HepG_2_ cells provided a more appropriate and translationally relevant system for high‐resolution flow cytometric analysis of apoptosis induced by *D*. *mucronata* extract and GND.

As illustrated in Figure 5, flow cytometry analysis was performed on HepG_2_ cells to evaluate apoptosis following treatment with *D*. *mucronata* extract and GND. The experiment included three groups: a control group (untreated cells), a *D*. *mucronata–*treated group (1.36 mg/mL), and a GND‐treated group (3.439 *μ*g/mL; 5.1 *μ*M). The rates of early apoptosis differed markedly among the groups. Control cells exhibited minimal early apoptosis (0.191%), whereas cells treated with *D*. *mucronata* showed a significantly higher early apoptotic rate of 64.2%, and GND‐treated cells displayed an early apoptotic rate of 61.4%. Similarly, late apoptosis increased substantially in the treated groups compared with the control group: 11.2% for *D*. *mucronata*, 24% for GND, and only 0.299% for the control. In contrast, no significant differences in necrosis rates were observed between the treated and control groups, indicating that both treatments primarily induced apoptotic rather than necrotic cell death.

**Figure 5:**
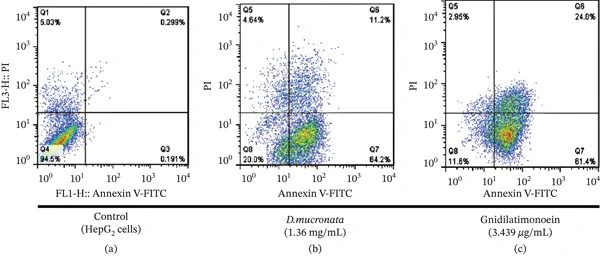
Apoptosis analysis conducted via flow cytometry using annexin V/PI staining in HepG_2_ cells. Apoptosis was analyzed in HepG_2_ cells using flow cytometry following annexin V‐FITC and PI double staining. Three experimental groups were examined: (a) untreated control cells, (b) cells treated with *D*. *mucronata* extract (1.36 mg/mL), and (c) cells treated with GND (3.439 *μ*g/mL). The flow cytometric plots display four distinct cell populations: viable cells (annexin V^−^/PI^−^; Q4), early apoptotic cells (annexin V^+^/PI^−^; Q3), late apoptotic cells (annexin V^+^/PI^+^; Q2), and necrotic cells (annexin V^−^/PI^+^; Q1). Both *D*. *mucronata* extract and GND treatments led to a substantial increase in early and late apoptotic cell populations compared with the control group, indicating activation of the apoptotic pathway rather than necrotic cell death. Abbreviations: GND, gnidilatimonoein; PI, propidium iodide; FITC, fluorescein isothiocyanate.

These findings demonstrate that both *D*. *mucronata* extract and GND effectively trigger apoptosis in HepG_2_ cells, with GND producing a stronger late‐apoptotic response. The predominance of apoptosis over necrosis suggests a targeted, regulated cell death mechanism rather than nonspecific cytotoxicity.

## 4. Discussion

In recent years, research on plant‐derived compounds has expanded significantly, driven by advances in cancer therapeutics and a growing interest in natural bioactive agents as safer alternatives to conventional chemotherapy [39, 40]. Consequently, there is increasing demand for effective medicinal formulations that utilize plant‐based compounds to inhibit tumor growth and metastasis [41, 42]. In 2011, Nirmala et al. [43] successfully isolated and characterized approximately 30 bioactive compounds from different plant species. Among these, natural products such as piperine, curcumin, and oleocanthal have demonstrated therapeutic potential against liver cancer, highlighting their relevance in oncological research [1]. Typically, this research begins with laboratory‐based investigations to assess efficacy and safety, progressing to preclinical and clinical evaluations when initial results are promising. In the present study, extracts from *D*. *mucronata* were prepared, and its purified diterpenoid compound, GND, was isolated. Both the crude extract and GND were evaluated for their effects on the proliferation of human and murine hepatocarcinoma cells.

Berberine, a well‐known plant alkaloid, has demonstrated encouraging antitumor activity across several cancer types. Recent studies investigating its combination with sorafenib—a standard therapeutic for HCC—revealed that the dual treatment significantly inhibited liver cancer cell growth (SMMC‐7721 and HepG_2_), in a dose‐ and time‐dependent manner, while increasing cleaved PARP and caspase‐3 levels, key indicators of apoptosis [44]. Similarly, an ethanolic extract of *Adenosma bracteosum* was reported to exhibit cytotoxic effects on HepG_2_ cells, with an IC_50_ value of 5.67 *μ*g/mL and activation of caspase‐3 [45].

In alignment with these findings, the present study demonstrated that both *D*. *mucronata* extract and GND significantly reduced the proliferation of HepG_2_ and Hepa1‐6 cells in a concentration‐dependent manner, with GND showing markedly lower IC_50_ values than the crude extract (see Results, Section 3.1), consistent with previous reports on the anticancer properties of plant‐derived diterpenoids [46–48]. Notably, although *D*. *mucronata* exhibited mild toxicity toward THLE‐2 cells at high concentrations, GND remained nontoxic, suggesting its potential for safer therapeutic use [49].

To further relate the cytotoxicity of the crude extract to its purified active constituent, we estimated the GND‐equivalent content within the extract′s IC_50_ dose, based on the measured GND yield of 1.71% (*w*/*w*) in the dry extract (Section 2.1). In HepG_2_ cells, the extract′s IC_50_ (1.36 mg/mL) corresponds to approximately 23.3 *μ*g/mL of GND‐equivalent content—roughly 6.8‐fold higher than the IC_50_ of purified GND alone (3.439 *μ*g/mL). This discrepancy was far more pronounced in Hepa1‐6 cells, where the extract′s IC_50_ (1.23 mg/mL) corresponds to approximately 21.0 *μ*g/mL GND‐equivalent, more than 600‐fold higher than the IC_50_ of purified GND in this cell line (34.23 ng/mL). This indicates that GND alone does not fully account for the cytotoxic potency of the crude extract, particularly in Hepa1‐6 cells, and suggests that either the bioavailability or activity of GND is reduced within the complex extract matrix, or that additional, as‐yet‐unidentified constituents of *D*. *mucronata* contribute independently to its anticancer activity. Importantly, this finding does not diminish the therapeutic relevance of GND itself; rather, it indicates that GND represents one of potentially several bioactive constituents contributing to the extract′s overall anticancer activity, and underscores GND′s value as a single, well‐characterized, highly potent, and selective compound suitable for standardized dosing—an advantage the structurally heterogeneous crude extract inherently lacks. This is consistent with the broader phytochemical literature indicating that *Daphne* extracts contain multiple bioactive diterpenoids beyond GND [6, 7], and highlights a direction for future bioassay‐guided fractionation studies to identify any additional active principles.

To quantify this differential toxicity, the SI was calculated for both agents (Table 1). Using a conservative approach, in which the highest tested concentration in THLE‐2 cells served as a lower bound estimate, the *D*. *mucronata* extract showed modest selectivity for malignant hepatocytes. Because its dose–response relationship in THLE‐2 cells remained statistically significant across the tested range, extrapolation of this regression was additionally justified and yielded substantially higher exploratory SI estimates exceeding the threshold conventionally associated with meaningful selectivity in the natural‐product cytotoxicity literature [50]. The convergence between these conservative and exploratory estimates strengthens confidence that the extract is genuinely, if moderately, selective toward cancer over normal hepatocytes rather than broadly cytotoxic.

The conservative SI calculated for GND was numerically lower than that of the extract (Table 1); however, this should not be interpreted as evidence that GND is inherently less selective. Rather, it reflects a methodological ceiling inherent to the present dataset: Because no statistically significant cytotoxicity trend was observed in THLE‐2 cells at any GND dose tested, extrapolation could not be justified, and GND′s true SI may be considerably higher than this conservative estimate. This asymmetry is further illustrated by the markedly larger conservative SI obtained for GND against Hepa1‐6 cells, although this comparison additionally involves a cross‐species contrast and should be interpreted with caution. Taken together, both agents show evidence of preferential activity against liver cancer cells over normal hepatocytes; although this could be quantified with greater confidence for the crude extract, the available data suggest that GND′s true selectivity is at least comparable to, and plausibly exceeds, that of the extract.

Sorafenib, an FDA‐approved treatment for advanced HCC, demonstrated an IC_50_ of 19.03 *μ*g/mL (40 *μ*M) in HepG_2_ cells (Figure 1g), consistent with earlier studies that reported IC_50_ values ranging between 4.81 and 39.4 *μ*g/mL [51, 52]. Although sorafenib effectively induces apoptosis and cell cycle arrest, its clinical use is limited by dose‐dependent toxicity and the emergence of drug resistance. The differences observed in IC_50_ values between HepG_2_ (human) and Hepa1‐6 (murine) cells can be attributed to variations in metabolic activity, receptor expression, and intrinsic sensitivity to cytotoxic agents. HepG_2_ cells, derived from human HCC, possess distinct signaling and drug metabolism pathways compared with the murine Hepa1‐6 line, resulting in species‐specific variations in drug response [53–55].

NO plays a complex and context‐dependent role in cancer biology, exerting both tumor‐promoting and tumor‐suppressive effects depending on its concentration, tumor type, and microenvironment [56, 57]. Elevated NO levels and increased expression of nitric oxide synthase (NOS) have been associated with poor prognosis in several cancers [58]. The liver is particularly sensitive to NO fluctuations, as chronic overproduction can contribute to DNA damage, inflammation, and hepatocarcinogenesis. Conversely, selective inhibition of NO synthesis has shown therapeutic potential in liver disease management. Previous studies reported that *D*. *mucronata* inhibits NO production in glial cells, exerting anti‐inflammatory effects [14]. Consistent with this, the current study found that both *D*. *mucronata* and GND significantly reduced NO levels in HepG_2_ and Hepa1‐6 cells (Figure 2), suggesting that suppression of NO production may contribute to their anticancer effects.

Alterations in intracellular biochemical markers further supported these findings. In clinical practice, elevated serum AST, ALT, ALP, LDH, and GGT reflect hepatocyte injury and enzyme leakage into the circulation, and these enzymes are frequently elevated in the serum of patients with liver malignancies due to increased tumor cell turnover and membrane damage [59, 60]. In the present in vitro system, intracellular enzyme activity in HepG_2_ and Hepa1‐6 cell lysates was instead used as a complementary marker of cellular integrity and metabolic status; treatment with *D*. *mucronata* extract and GND significantly decreased intracellular activity of all five enzymes in a dose‐dependent manner (Tables 2 and 3). Given that these enzymes are principally cytosolic, this decline most plausibly reflects progressive loss of plasma membrane integrity and consequent enzyme leakage into the extracellular medium—the same principle underlying established enzyme‐release cytotoxicity assays such as the LDH‐release assay [34, 35]. This interpretation is consistent with the cytotoxic and proapoptotic effects observed by MTT and flow cytometry, rather than representing a direct intracellular parallel of the serum elevations seen in clinical HCC. This distinction should be borne in mind when extrapolating these in vitro findings to the clinical biomarker context.

Apoptosis is a fundamental mechanism for maintaining cellular homeostasis, and its evasion is a hallmark of cancer [61, 62]. Caspase‐3 serves as a crucial executioner enzyme in this process, cleaving structural and regulatory proteins during programmed cell death [63]. In this study, both *D*. *mucronata* and GND markedly increased caspase‐3 activity in HepG_2_ and Hepa1‐6 cells (Figure 3), confirming their ability to induce apoptosis.

To elucidate the underlying molecular mechanisms, Western blot analysis was conducted on GND‐treated HepG_2_ cells (Figure 4). GND was chosen for this assay due to its greater potency, selectivity, and lower cytotoxicity compared with the crude extract. The results showed significant upregulation of proapoptotic proteins Bax, P53, and cleaved caspase‐3, alongside downregulation of the antiapoptotic protein Bcl‐2, resulting in a marked shift in the Bax/Bcl‐2 ratio consistent with engagement of the intrinsic (mitochondrial) apoptotic pathway, in which an elevated Bax/Bcl‐2 ratio is classically associated with mitochondrial outer membrane permeabilization, cytochrome c release, and caspase‐9–dependent activation of caspase‐3 [23, 64–69]. This pattern closely parallels findings reported for other plant‐derived diterpenoids and terpenoid‐class natural compounds in HepG_2_ cells. For instance, the clerodane diterpenoid mallotucin D and the sesquiterpene lactone alantolactone similarly induce HCC cell death through coordinated Bax upregulation, Bcl‐2 suppression, and caspase‐3 activation [70, 71], Dose‐dependent upregulation of Bax and caspase‐9, with concomitant Bcl‐2 downregulation, has also recently been reported for the plant‐derived agent *Woodfordia fruticosa* in HepG_2_ cells [72], underscoring the Bax/Bcl‐2‐caspase‐3 axis as a convergent mechanism across structurally diverse natural anticancer compounds. Notably, this mechanism also appears conserved within the *Daphne* genus itself: A methanolic extract of *D*. *mucronata* has recently been shown to induce MCF‐7 breast cancer cell apoptosis through a mitochondria‐dependent, caspase‐mediated pathway, with molecular docking indicating preferential binding of its active diterpenoid to Bax over Bcl‐2 [73], whereas structurally related daphnane‐type diterpenoids from *Daphne genkwa* and *Daphne pontica* similarly elevate the Bax/Bcl‐2 ratio and activate caspase‐3/9 in colon and prostate cancer cell lines, respectively [74, 75]. These findings collectively support a shared, genus‐conserved mechanism of mitochondrial apoptosis induction by *Daphne*‐derived diterpenoids across diverse cancer cell types, extending the present findings in hepatocellular carcinoma to a broader pharmacological context. However, because cytochrome c release, mitochondrial membrane potential (e.g., by JC‐1 staining), and intracellular ROS levels were not directly measured in the present study, this interpretation should be regarded as inferential rather than directly demonstrated. Direct confirmation of mitochondrial outer membrane permeabilization and its downstream events represents an important objective for future mechanistic studies.

Flow cytometry results provided additional confirmation of apoptosis induction, with both *D*. *mucronata* and GND producing substantial increases in early and late apoptotic populations relative to control cells (Figure 5; see Results, Section 3.6, for exact percentages). Minimal necrosis was detected, indicating that the observed cytotoxicity resulted primarily from programmed cell death rather than nonspecific necrotic damage. This magnitude of apoptosis induction, and the predominance of apoptotic over necrotic cell death, is consistent with recent reports on other plant‐derived agents in HepG_2_ cells; for instance, *W*. *fruticosa* extract has similarly been shown to produce a dose‐dependent increase in the apoptotic cell population (from 7.6% in untreated controls to approximately 55% at the highest tested dose), accompanied by coordinated Bax/caspase‐9 upregulation and Bcl‐2 downregulation [72]. This reinforces the Bax/Bcl‐2‐caspase axis as a common mechanistic route by which structurally diverse natural compounds induce apoptotic, rather than necrotic, cell death in HCC cell lines. HepG_2_ cells were selected for this assay because they represent a clinically relevant human model with well‐characterized apoptotic signaling pathways.

Several practical and translational considerations warrant discussion. The pharmacokinetic profile of GND and the crude extract—including their stability, aqueous solubility, systemic bioavailability, and delivery to hepatic or tumor tissue following in vivo administration—have not yet been characterized and remain an essential prerequisite for any translational development. Likewise, the host metabolism, potential immunogenicity, and off‐target effects of GND, as well as its possible pharmacokinetic or pharmacodynamic interactions with chemotherapeutic, endocrine, or immunotherapeutic agents, have not been investigated and will require dedicated in vivo and preclinical safety pharmacology studies. Regarding toxicity, it is well documented that plants of the *Daphne* genus, including species closely related to *D*. *mucronata*, contain known toxic diterpenoid constituents such as daphnetoxin and mezerein, which have been associated with gastrointestinal, dermal, and, in severe cases, systemic toxicity following ingestion or contact with crude plant material [6]. The present study found that both the crude extract and, in particular, purified GND showed minimal cytotoxicity toward normal THLE‐2 hepatocytes in vitro, supporting a degree of selectivity at the cellular level. However, this does not establish systemic safety and formal toxicological evaluation—such as acute and repeated‐dose toxicity studies in animal models—will be essential before GND′s therapeutic potential can be meaningfully assessed.

Nevertheless, several limitations should be acknowledged. With respect to study design, this work was conducted using only two liver cancer cell lines, which may limit the generalizability of the findings to other HCC subtypes or cancers of different origins. Moreover, although in vitro systems offer controlled environments for mechanistic study, they cannot fully replicate the complexity of in vivo tumor–host interactions. Future work should therefore incorporate animal models, primary human liver cancer cells, and longer exposure periods to assess chronic effects and potential resistance mechanisms.

With respect to mechanistic depth, the present study focused specifically on apoptosis and cytotoxicity; other pathways—including oxidative stress, autophagy, and immune modulation—may also contribute to GND′s anticancer effects and warrant further investigation. Similarly, our conclusion of intrinsic (mitochondrial) pathway engagement relied on Bax, Bcl‐2, P53, and caspase‐3 expression as indirect markers; direct functional evidence, including cytochrome c release, mitochondrial membrane potential via JC‐1, and intracellular ROS, was not obtained. In addition, a parallel chemically induced positive apoptotic control (e.g., staurosporine, doxorubicin, or sorafenib) was not included across the Western blot, flow cytometry, and caspase‐3 assays to directly benchmark assay sensitivity. Complementary assays such as a combined caspase‐3/7 activity assay and cell‐cycle analysis with sub‐G1 quantification would provide further orthogonal confirmation and are recommended for future work.

With respect to quantitative precision, because IC_50_ in THLE‐2 cells was not reached within the tested range, the reported selectivity indices (Table 1) represent conservative or extrapolated estimates rather than precisely measured values, particularly for GND. Likewise, AST, ALT, ALP, LDH, and GGT activities were measured in intracellular lysates rather than culture supernatants or serum, which, although a useful index of cytosolic enzyme retention, does not directly replicate serum‐based clinical interpretation. Finally, triplicate measurements across the downstream biochemical, caspase‐3, and Western blot assays represented technical replicates from cultures of the same passage and day rather than independent biological replicates across separate passages; incorporating true biological replication in future studies would further strengthen confidence in the reproducibility of the reported effect magnitudes.

Finally, several practical translational considerations—including pharmacokinetics, stability, solubility, bioavailability, tissue delivery, host metabolism, immunogenicity, potential drug interactions, and the known toxicity of Daphne‐derived diterpenoids—remain unaddressed by the present in vitro study and represent essential priorities for future preclinical development before GND′s therapeutic potential can be meaningfully assessed.

## 5. Conclusions

This in vitro study highlights the antiproliferative and proapoptotic effects of *D*. *mucronata* extract and its purified compound, GND, against liver cancer cell lines, specifically HepG_2_ and Hepa1‐6. The results demonstrate that these compounds not only suppress cell viability but also modulate key apoptosis‐regulatory proteins, as evidenced by increased caspase‐3 activity and a shift in the Bax/Bcl‐2 ratio consistent with engagement of the intrinsic apoptotic pathway. Importantly, GND displayed a nontoxic profile toward THLE‐2 cells, underscoring its potential as a safer and more selective therapeutic candidate.

Although previous studies have documented the anticancer properties of various plant‐derived compounds, the present findings provide new evidence supporting the therapeutic promise of *D*. *mucronata* and GND in the treatment of hepatocellular carcinoma. Because these findings are derived exclusively from cell culture models, their clinical and translational relevance remains speculative and should be regarded as preliminary until confirmed through in vivo studies in appropriate animal models of hepatocellular carcinoma, and eventually through clinical evaluation. Collectively, this research establishes a foundation for future investigations into the molecular mechanisms and clinical applicability of *D*. *mucronata*, advancing the broader development of plant‐based compounds as effective and less toxic alternatives in cancer therapy.

**Nomenclature**
ALP: alkaline phosphatase
ALT: alanine aminotransferase
ANOVA: analysis of variance
APAC‐1: apoptotic protease activating factor 1
AST: aspartate aminotransferase
BCA: bicinchoninic acid
DAB: 3, 3 ^′^‐diaminobenzidine
DMEM: Dulbecco′s Modified Eagle Medium
DMSO: dimethyl sulfoxide
ECL: enhanced chemiluminescence
FBS: fetal bovine serum
FITC: fluorescein isothiocyanate
GAPDH: glyceraldehyde‐3‐phosphate dehydrogenase
GGT: gamma glutamyl transferase
GND: gnidilatimonoein
HPLC: high‐performance liquid chromatography
LDH: lactate dehydrogenase
MOMP: mitochondrial outer membrane permeabilization
MTT: 3‐(4,5‐dimethylthiazol‐2‐yl)‐2,5‐diphenyltetrazolium bromide
NaNO_2_
: sodium nitrite
NO: nitric oxide
NOS: nitric oxide synthase
PI: propidium iodide
PMSF: phenylmethylsulfonyl fluoride
SDS‐PAGE: sodium dodecyl sulfate–polyacrylamide gel electrophoresis
SEM: standard errors of the means
TACE: transarterial chemoembolization

## Author Contributions

Sevda Zarei: methodology, performing the experiments, writing—original draft, and statistical analysis. Manijeh Mianabadi: conceptualization, supervision, methodology, and writing—review and editing. Mahnaz Khalafi: adviser, statistical analysis, and validation.

## Funding

This study was supported by the Golestan University (10.13039/100020925; 9111023504).

## Disclosure

The authors take full responsibility for the content of the published article.

## Ethics Statement

No ethics approval was required, as confirmed by the institutional guidelines of the Pasteur Institute of Iran and national regulations on biomedical research using established cell lines (National Biomedical Research Ethics Code, Ministry of Health and Medical Education, Iran, 2014).

## Consent

This study utilized commercially available, deidentified cell lines and did not involve human participants, human data, or human tissue. All authors have agreed to the submission and publishing.

## Conflicts of Interest

The authors declare no conflicts of interest.

## General Statement

*Cell Line Authentication and Validation Statement*. The human hepatocellular carcinoma cell line HepG_2_ (human and liver tissue; sex information was not available from the supplier′s documentation; RRID: CVCL_0027), the murine hepatocarcinoma cell line Hepa1‐6 (mouse and liver tissue; sex information was not available from the supplier′s documentation; RRID: CVCL_0327), and the normal human hepatocyte cell line THLE‐2 (human, liver tissue; sex information was not available from the supplier′s documentation; RRID: CVCL_0415) were used in this study. All cell lines were obtained from the National Cell Bank of Iran (NCBI), Pasteur Institute of Iran in April 2023. According to the documentation provided by the supplying cell bank, the deposited cell lines had undergone routine authentication procedures prior to distribution, including short tandem repeat (STR) profiling for the human cell lines and mycoplasma contamination testing. The exact STR match percentage was not available to the authors from the supplier′s documentation. No additional independent authentication was performed by the authors beyond the certification provided by the supplying cell bank. The cell lines used in the experiments were reported by the supplier to be free of mycoplasma contamination at the time of distribution. All cell lines were used within a limited passage number following resuscitation, and periodic morphological inspection was performed throughout the study to monitor the absence of contamination and phenotypic drift.

## Supporting information


**Supporting Information** Additional supporting information can be found online in the Supporting Information section. The original Western blot images are provided in the Supporting Information file. The supplementary materials provide additional methodological details and experimental data supporting the findings presented in the main manuscript. They include information on the extraction of *D*. *mucronata*, the chromatographic analysis and purification of GND, total protein measurements, and the original Western blot images used for protein expression analysis. Supplementary Figure 1: (a–e) Characterization of the extraction and purification procedures for *D*. *mucronata* and GND. The UV absorption spectra of ethanol and the aqueous plant extract are presented in Supplementary Figure 1a,b, respectively. Supplementary Figure 1c shows the HPLC chromatogram of the GND standard, whereas Supplementary Figure 1d presents the chromatographic profile of the plant extract, showing a retention time of 16.4 min for the peak corresponding to GND. The chemical structure of GND is provided in Supplementary Figure 1e. The chromatographic conditions and column specifications are also provided in the supplementary data. Supplementary Figure 2: (a,b) Total protein content in HepG_2_ and Hepa1‐6 cells, respectively, as determined by the Bradford assay. The data are presented as mean ± SEM. Different letters indicate statistically significant differences between experimental groups, as determined by Tukey′s HSD post hoc test (*p* < 0.01). Supplementary Figures 3, 4, 5, 6, and 7: Original Western blot images supporting the protein expression analyses presented in the main manuscript. These images include Bax (Supplementary Figure 3), Bcl‐2 (Supplementary Figure 4), P53 (Supplementary Figure 5), caspase‐3 (Supplementary Figure 6), and GAPDH as the loading control (Supplementary Figure 7). Where applicable, both initial optimization experiments and the blots used for quantitative analysis are provided. These images are included to document the original experimental data underlying the reported protein expression results.

## Data Availability

The data that support the findings of this study are available from the corresponding author upon reasonable request.
