# Supplementary material for: Apoptosis‐Inducing and Cytotoxic Effects of the Daphne mucronata Extract and Its Bioactive Compound Gnidilatimonoein in Liver Cancer Cells
Source: Biomed Res Int. 2026 Nov 14;2026:3638482. doi: 10.1155/bmri/3638482 (PMC13768926; doi:10.1155/bmri/3638482)
Supplement: Supplementary file 1 [file BMRI-2026-3638482-s001.docx]

***1. Supplementary data***

***1.1. Steps for extracting D. mucronata and purifying GND***

***1.1.1. Ethanol extraction of D. mucronata***

To ensure that the extract solution was free of ethanol and to evaluate its impact on cells, spectrophotometry was used. initially, the absorbance of ethanol obtained from Merck was measured at wavelengths ranging from 200 to 240 nm using a spectrophotometer (Fig. 1a). Subsequently, the absorbance of the diluted extract in water was assessed (Fig. 1b).

**Figure 1a. UV spectrum of ethanol.**

**Figure 1b. UV spectrum of *D. mucronata* extract.**

***1.1.2. Conditions for column chromatography for GND purification***

The chromatography column was prepared by filling it with diethyl ether after mixing silica gel (130–270 mesh size; Merck) with the appropriate solvent mixture to achieve equilibrium between the two phases. Diethyl ether was then used to wash the chromatographic column, ensuring that equilibrium was maintained. The specifications of the column utilized for separating the active ingredient from the plant extract are detailed in Table 1.

**Table 1. Specifications of the chromatography column.**

| Column length | 40 cm |
| --- | --- |
| Column inner diameter | 1.5 cm |
| Height of silica gel in the column | 35 cm |
| Flow rate | One drop in 5 seconds |

***1.1.3. Analysis of pure GND using HPLC***

Initially, the active compound GND (Mianabadi et al., 2004; Salehloo et al., 2023) was dissolved in ethanol to serve as a standard. To identify this compound using the HPLC system, the analysis was conducted at a wavelength of 326 nm, with a flow rate of 1 mL/min, utilizing a Zorbax 18C column. The mobile phase consisted of deionized water and acetonitrile, and the injection program followed the gradient outlined in Table 2.

**Table 2. Gradient flow program of the mobile phase in HPLC.**

| Acetonitrile (%) | Time (min) |
| --- | --- |
| 0 | 5 |
| 5 | 15 |
| 25 | 45 |
| 30 | 50 |

Taking into account withdrawal time, the GND compound was purified after injecting 500 µL of concentrated *D. mucronata* extract via HPLC (Fig 1c and 1d). The GND structure is also shown in Fig 1e.

**Figure 1c. Chromatogram of GND obtained using Agilent A1260 HPLC system.** The HPLC conditions consisted of a mobile phase comprising deionized water and an acetonitrile gradient, with a flow rate of 1 mL/min and a detection wavelength of 326 nm.

**Figure 1d.** Analytical chromatogram of *D. mucronata* extract, showing that the retention time for the GND compound is 16.4 minutes, consistent with the retention time observed for the purified standard (Fig. 1c)**.**

**Figure 1e. Chemical structure of GND.**

***1.2. Total protein***

The Bradford assay revealed a similar pattern in both HepG_2_ and Hepa 1-6 cells (Fig. 2; *P* ˂ 0.01).

**Figure 2a. Total protein content in HepG_2_ cells.** The vertical bars represent the standard error (mean ± SEM), and different letters within each column indicate statistically significant differences among groups as determined by Tukey's HSD post hoc test (a, b, c, etc.; *P* < 0.01).

**Figure 2b. Total protein content in Hepa 1-6 cells.** The vertical bars denote the standard error (mean ± SEM), with different letters in each column indicating significantly different groups according to Tukey's HSD post hoc test (a, b, c, etc.; *P* < 0.01).

***1.3. Original images of the western blot gel***

Images depicting the protein expression levels of Bax, Bcl-2, P53 and caspase-3 in HepG_2_ cells are presented in Sections 2-5 and Figs. 3-7.

**a)**

**b)**

**Figure 3. Original images of the Western blot gel for Bax.** Two independent gel runs are shown: (a) an initial optimization run (control and GND-treated lanes, run in duplicate) performed to verify antibody specificity and signal detection prior to the main experiment; (b) the gel used for the quantitative analysis presented in Fig. 4 of the main text (control vs. GND-treated, 5.1 µM).

**a)**

**b)**

**Figure 4. Original images of the Western blot gel for Bcl-2.** Two independent gel runs are shown: **(a)** an initial optimization run (control and GND-treated lanes, run in duplicate) performed to verify antibody specificity and signal detection prior to the main experiment; **(b)** the gel used for the quantitative analysis presented in Fig. 4 of the main text (control vs. GND-treated, 5.1 µM).

**a)**

**b)**

**Figure 5. Original images of the Western blot gel for P53.** Two independent gel runs are shown: **(a)** an initial optimization run (control and GND-treated lanes) performed to verify antibody specificity and signal detection prior to the main experiment; **(b)** the gel used for the quantitative analysis presented in Fig. 4 of the main text (control vs. GND-treated, 5.1 µM).

**Figure 6. Original image of the Western blot gel for Caspase-3.** The image shows two independent replicate lanes for each group (control and GND-treated, 5.1 µM) run side-by-side on the same membrane, flanking the molecular weight marker.

**a)**

**b)**

**Figure 7. Original images of the Western blot gel for GAPDH (loading control).** Two independent gel runs are shown: **(a)** an initial optimization run (control and GND-treated lanes, run in duplicate) performed to verify antibody specificity and signal detection prior to the main experiment; **(b)** the gel used for the quantitative analysis presented in Fig. 4 of the main text (control vs. GND-treated, 5.1 µM).
